# Health Impairment Notifications About Doctors to the Australian Medical Regulator, 2012–2022: A Retrospective Cohort Study

**DOI:** 10.5694/mja2.70131

**Published:** 2026-01-14

**Authors:** Marie M. Bismark, Dilanka Hettiarachchi, Martin Fletcher, Owen Bradfield, Anu Tayal, Yamna Taouk

**Affiliations:** ^1^ Centre for Health Policy, Melbourne School of Population and Global Health The University of Melbourne Melbourne Victoria Australia; ^2^ RMIT University Melbourne Victoria Australia; ^3^ Medical Indemnity Protection Society Melbourne Victoria Australia

**Keywords:** doctor's health, impairment, patient safety

## Abstract

**Objectives:**

To assess the prevalence, characteristics and outcomes of health impairment notifications to the Australian Health Practitioner Regulation Agency (Ahpra) and to assess the influence of doctor age, sex, specialty, practice location and country of training on the incidence of health impairment notifications.

**Study Design:**

Retrospective cohort study; analysis of linked de‐identified Ahpra medical register and health impairment notifications data.

**Setting, Participants:**

All doctors registered to practise in Australia (except New South Wales) for whom notifications of concerns about physical or mental illness, cognitive decline, substance use disorder or other impairment to safely practising medicine were received by Ahpra during 1 July 2012–30 June 2022.

**Main Outcome Measures:**

Health impairment notifications, overall and by notification type and specialty; influence of doctors' characteristics on the incidence of notifications.

**Results:**

During 2012–2022, 112,677 doctors were registered to practise in Australia (other than New South Wales). A total of 1732 health impairment notifications were recorded, including at least one notification for 1258 doctors (1.1%). In multivariable analyses, the incidence of health impairment notifications was higher for male than female doctors (adjusted incidence rate ratio [aIRR], 1.45; 95% confidence interval [CI], 1.26–1.67), for doctors aged 70 years or older than for those aged 30–39 years (aIRR, 2.92; 95% CI, 2.30–3.70) and for doctors in regional (aIRR, 1.33; 95% CI, 1.12–1.58), rural (aIRR, 1.27; 95% CI, 1.03–1.57) and remote areas (aIRR, 1.55; 95% CI, 1.03–2.33) than in metropolitan areas. Among doctors with specialist qualifications, the incidence of notifications was higher for psychiatrists than internal medicine physicians (aIRR, 2.28; 95% CI, 1.62–3.21) and the incidence of substance use notifications was highest for anaesthetists (vs. internal medicine physicians: aIRR, 2.83; 95% CI, 1.66–4.83). Compared with doctors who trained in Australia, doctors who trained in non‐comparable jurisdictions were less likely to be subjects of health impairment notifications (aIRR, 0.53; 95% CI, 0.43–0.64). Of 1708 notifications with final Ahpra determinations, 367 (21.5%) resulted in practice restrictions or removal from practice.

**Conclusions:**

Health impairment notifications are infrequent but can have serious consequences for doctors. The incidence of health impairment notifications is influenced by doctor age, sex, specialty and location. Specific measures that take these factors into account could support workplace health and safety for doctors and protect patients from harm.

## Plain Language Summary

**The known**: Fear of regulatory involvement may deter unwell doctors from seeking help, but health care regulators must protect the public by ensuring that doctors are fit to practise.

**The new**: During 2012–2022, the medical regulator received notifications of concern about health impairments for 1% of doctors in Australia. Reports were more frequent for male doctors, doctors aged 70 years or older, psychiatrists and general practitioners. Disproportionate numbers of anaesthetists and rural doctors were subjects of substance use notifications. The medical regulator imposed practice restrictions, such as conditions or suspension, following 21.5% of notifications.

**The implications**: Responses to reports of impairment in doctors should be targeted and evidence‐based, providing support for doctors while protecting patients.

When doctors with a mental or physical illness, cognitive decline or substance use disorder are impaired and place the public at risk of harm, medical regulators may need to intervene. Importantly, having a health condition does not necessarily mean that a doctor is impaired. Most unwell doctors keep their patients safe by seeking treatment, taking leave or adjusting their practice. However, if the safe practice of medicine is compromised by a doctor's health condition, the Australian Health Practitioner Regulation Agency (Ahpra) must be notified [1]. The Medical Board of Australia may then take regulatory action, such as requiring a health assessment, imposing conditions on practice or, rarely, removing a doctor from practice.

Concern about doctors' health has been reflected in decades of scholarship and professional activity [2, 3, 4, 5]. The introduction of mandatory reporting of impaired doctors in 2010 [6] and a recent proposal in Australia that older doctors undergo health checks sparked robust debate [7, 8]. Some members of the profession fear that regulatory involvement could deter unwell doctors from seeking help and that the stress of regulatory processes could delay recovery or even precipitate suicidal behaviour [9, 10, 11]. In response, regulators are seeking to adopt more compassionate [12] and evidence‐based approaches to protecting the public while supporting safe medical practice. In December 2025, the Medical Board announced that it would trial profession‐led support for older doctors, rather than proceeding with mandatory health checks [13].

The prevalence of impairment among doctors in Australia is unknown. Limited epidemiological evidence from overseas suggests that as many as 14% of doctors are impaired at some point in their careers [14, 15]. In the United States, it is estimated that each year, about 3% of doctors experience health conditions sufficiently severe to cause impairment [16, 17].

Notifications to Ahpra that a doctor may be impaired (health impairment notifications) can be made voluntarily by any person or under mandatory reporting laws that apply to employers and other health practitioners (Table 1). Impaired doctors may also report themselves. Despite these requirements, it is unlikely that all impaired doctors come to the attention of regulators [14, 15, 16, 17].

**TABLE 1:** Mandatory notification of health impairment of medical practitioners to Australian health profession regulators [18].

Who must make a mandatory notification?	Registered health practitioners, employers and educators have a legal duty to make a mandatory notification in certain circumstances.
Who can make a voluntary notification?	Anyone, including patients and members of the public, can make a voluntary notification.
Who is exempt from reporting?	Practitioners who are providing legal advice, indemnity insurance or are engaged in certain quality assurance functions.
What are the grounds for reporting health concerns?	An employer or registered health practitioner (other than a treating practitioner) must form a reasonable belief that a doctor is practising while intoxicated by alcohol or drugs, or is practising with an impairment which places the public at risk of substantial harm. A treating practitioner must form a reasonable belief that a doctor is practising while intoxicated by alcohol or drugs or is practising with an impairment, which places the public at substantial risk of harm.
What factors are relevant in assessing the risk of harm?	The nature and severity of the condition, the practice type, the extent to which the condition can be managed by treatment or other strategies and the doctor's level of insight and engagement with treatment.
When is notification not required?	A notification is not required if there are effective controls for managing the impairment and reducing the risk and severity of harm to the public. These controls may include treatment, a break from practice, such as sick leave, modified scope of practice or compliance with monitoring and supervision.
What about treating practitioners?	Treating practitioners in Western Australia are exempt from making mandatory reports (but may still have an ethical responsibility to make a voluntary report). Treating practitioners in other states have a higher threshold for reporting than non‐treating practitioners (see above).
How does the law protect notifiers?	Anyone who makes a notification in good faith is protected from liability under the *Health Practitioner Regulation National Law*.

We examined 10 years of Ahpra registration and notifications data to assess the prevalence, characteristics and outcomes of health impairment notifications, and how risk varies by sex, age, practice location, country of training and medical specialty. Our aim was to facilitate the identification of doctors at risk of impairment in order to support their return to safe practice, thereby protecting both doctors and the public from harm.

## 1 Methods

We undertook a retrospective cohort study. Ahpra provided de‐identified quantitative data for all health impairment notifications about doctors lodged during 1 July 2012–30 June 2022. As there is a lag between receipt of notifications and final Medical Board decisions, data were extracted in June 2024, allowing time for notifications to be investigated and outcomes to be decided. We report our study in accordance with the Strengthening the Reporting of Observational Studies in Epidemiology (STROBE) guideline [19].

### 1.1 Data Sources

De‐identified, routinely collected administrative data were provided by Ahpra, the agency responsible for regulating 16 health professions in Australia. Our study included all notifications for all registered doctors in all Australian states and territories other than New South Wales. Ahpra does not receive or investigate notifications from New South Wales; Queensland also has a different regulatory model, but Ahpra collects sufficient notifications data from Queensland to include them in our analysis. We also excluded doctors registered to addresses outside Australia throughout the study period.

Ahpra provided de‐identified demographic data for all doctors registered in Australia during 2012–2022: age, sex, medical specialty, jurisdiction, practice location (geographic remoteness), country of training and duration of registration.

Ahpra provided unique identification numbers that facilitated linkage of notifications data with registrations data for this study.

### 1.2 Variables

We coded doctors into seven specialty categories: internal medicine, general practice, surgery, psychiatry, anaesthesia, other specialties and non‐specialists (including recent graduates, doctors in specialty training and doctors working in other roles with general and limited registration). Doctors were grouped into 10‐year age bands and moved bands as they aged. Doctors were recorded as female or male according to self‐identified sex at baseline.

Country of training, based on registration pathways used by Ahpra, was categorised as Australia, comparable jurisdictions (New Zealand, Canada, the United States, the United Kingdom and Ireland) [20] and non‐comparable jurisdictions. Remoteness was classified according to the Modified Monash Model [21], collapsed into four categories (metropolitan, regional, rural and remote).

Using categories provided by Ahpra, we coded the nature of each health impairment notification according to whether it primarily related to physical health, mental health, substance use, cognition (Table 2) or other. Notifications about performance or conduct concerns were not included in our analysis. For cases that involved more than one category, we selected one only, giving priority to organic causes of illness.

**TABLE 2:** Types of health impairment notification about doctors to Australian health profession regulators: Examples.

Type of impairment	Example and outcome
Mental illness	A psychiatrist working in a community mental health clinic makes a voluntary self‐report that she has been diagnosed with post‐traumatic stress disorder related to several episodes of occupational violence. The psychiatrist is receiving ongoing treatment from a psychologist and psychiatrist, who both provide reports to the Medical Board stating that the psychiatrist is taking sick leave when needed, is following all treatment recommendations, and that they have no concerns about her fitness to practise. As there is no identifiable risk to the public, the Board decides to take no further action.
Physical illness	A nurse makes a mandatory report that a surgeon is losing his hearing and has made several errors at work because he misunderstands conversations in the operating theatre. The surgeon provides a report from his audiologist confirming moderate hearing loss that has been corrected with a hearing aid. The Board accepts a voluntary undertaking from the surgeon that he will undergo regular hearing tests, will use hearing aids at work, and will advise his employer of his hearing loss so that appropriate support can be provided.
Substance use	An anaesthetist uses fentanyl recreationally. His use is becoming more frequent, and his employer makes a mandatory report that he has misappropriated fentanyl intended for patient use and has used fentanyl at work. The Medical Board requires the anaesthetist to undergo a health assessment and suspends the anaesthetist pending the outcome of an investigation. Following the investigation, including assessment of reports from treating practitioners, the Board imposes conditions that require him to work under supervision and to undergo random drug tests.
Cognitive impairment	An older general practitioner is diagnosed with dementia. He works as a sole practitioner in a busy rural practice and insists that his memory loss is not affecting his practice. A local pharmacist reports repeated concerns about his prescribing practices, which include incorrectly prescribing medications and doses. The general practitioner is unwilling to consider options such as working under supervision in a nearby group practice and refuses to undergo a health assessment. The Medical Board suspends the practitioner. The general practitioner seeks advice from his indemnity insurer, who supports him in deciding it is time to retire.

We coded notification sources as employer, treating practitioner, other health practitioner, the doctor themselves (self‐report), patients or their representative or other. We grouped concerns raised by patients and families, whether directly with Ahpra or via complaint bodies [22] and notifications from other community members, as notifications received from patients or their representatives.

The outcome was coded according to the final determination. ‘No further action’ meant that either the impairment allegation was not substantiated or an intervention to protect the public was not required, for example, when the health condition had resolved or was being appropriately managed. We used the term ‘restrictive action’ when the Medical Board or a tribunal made a final determination to restrict a doctor's practice by imposing conditions (such as drug testing), suspension or cancellation of registration. Voluntary undertakings are conditions agreed between the doctor and the Medical Board.

### 1.3 Statistical Analyses

We structured the dataset using a person–period format in which each doctor's registration interval was split into distinct one‐year intervals. This allowed us to capture risk dynamically across time. To control for general changes and policy changes over time, we included calendar year of registration (time‐varying for each year that the doctor was included in the study) as a categorical variable.

We report the characteristics of doctors and the nature of health concerns as counts and proportions. For doctors for whom at least one health impairment notification was recorded, we used the demographic data at the time of the first notification. For doctors for whom no health impairment notifications were recorded, we used the demographic data for their first entry in the dataset.

The primary outcome was a health impairment notification, coded as 1 for each year in which at least one or more health impairment notification was recorded for a registered doctor, and 0 for each year in which no health impairment notification was recorded.

We examined associations between demographic characteristics and health impairment notifications using multivariate logistic regression analysis. We estimated the adjusted incidence rate ratio (aIRR) with 95% confidence interval (CI) for health impairment notifications by variable over time in generalised linear models with Poisson distribution and log‐link function, and robust estimators of variance clustered by identification number. Cluster‐robust standard errors were used to account for any correlation of repeated notifications for individuals over time. We repeated the analysis for substance use notifications only. In a sensitivity analysis, we included only health impairment notifications that resulted in regulatory action.

All analyses were conducted in Stata 18.0.

### 1.4 Ethics Statement

The study was approved by the Human Research Ethics Committee of the University of Melbourne (reference number 22933). Ahpra provided de‐identified data under a deed of confidentiality and a strict data protection plan.

## 2 Results

**FIGURE 1:**
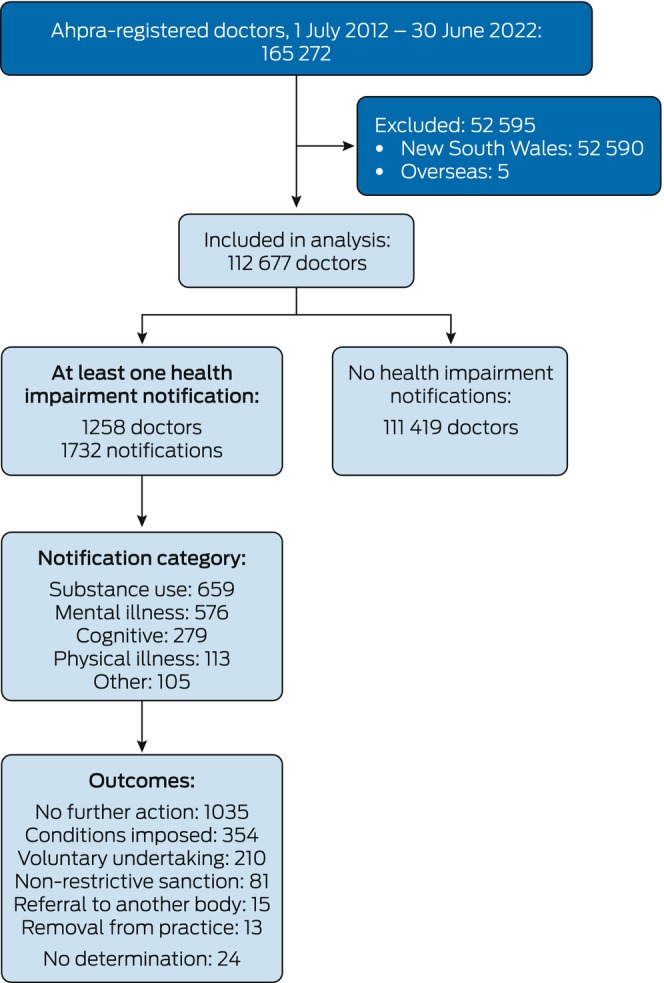
Notification of health impairment of medical practitioners to Australian health profession regulators, Australia (except New South Wales), 1 July 2012–30 June 2022. Ahpra, Australian Health Practitioner Regulation Agency.

During 1 July 2012–30 June 2022, 112,677 doctors were registered to practise in Australia (excluding 52,590 in New South Wales and five with overseas addresses); the median duration of registration was 9 years (interquartile range, 4–10 years). At least one health impairment notification was recorded for 1258 doctors (1.1%); a total of 1732 health impairment notifications were recorded (Figure 1). Thirty‐three of 1732 notifications (1.9%) raised more than one health concern.

The five specialty groups including the largest numbers of doctors for whom notifications were recorded were general practice (417 doctors, 33.6%), surgery (121, 9.7%), internal medicine (91, 7.3%), anaesthesia (69, 5.6%) and psychiatry (64, 5.2%); 434 notifications (34.9%) concerned non‐specialists (Table 3).

**TABLE 3:** Characteristics of all practising doctors and of doctors for whom health impairment notifications were recorded, Australia (except New South Wales), 1 July 2012–30 June 2022.

Characteristic	All doctors	Doctors with health impairment notifications
Number of doctors	112,677	1258
Sex		
Male	62,995 (55.9%)	865 (68.8%)
Female	49,679 (44.1%)	393 (31.2%)
Missing data	3	0
Age group (years)		
Under 30	33,085 (29.4%)	65 (5.2%)
30–39	33,155 (29.4%)	255 (20.3%)
40–49	20,239 (18.0%)	286 (22.7%)
50–59	14,101 (12.5%)	295 (23.4%)
60–69	8279 (7.4%)	196 (15.6%)
70 or older	3818 (3.4%)	161 (12.8%)
Specialty		
General practice	18,735 (17.7%)	417 (33.6%)
Surgery	5872 (5.5%)	121 (9.7%)
Internal medicine	7271 (6.9%)	91 (7.3%)
Anaesthesia	3744 (3.5%)	69 (5.6%)
Psychiatry	2645 (2.5%)	64 (5.2%)
Other specialty	5243 (5.0%)	46 (3.7%)
Non‐specialist	62,298 (58.9%)	434 (34.9%)
Missing data	6869	16
Location		
Metropolitan	83,239 (78.8%)	921 (74.4%)
Regional	13,661 (12.9%)	171 (13.8%)
Rural	7446 (7.0%)	118 (9.5%)
Remote	1347 (1.3%)	28 (2.3%)
Missing data	6984	20
Country of training		
Australia	69,110 (63.8%)	875 (74.1%)
Comparable jurisdictions	17,399 (16.1%)	139 (11.8%)
Non‐comparable jurisdictions	21,840 (20.2%)	167 (14.1%)
Missing data	4328	77

### 2.1 Distribution of Health Concern Types

During 2012–2022, male doctors comprised 55.9% of the medical workforce included in our analysis (Table 3); 865 of 1258 health impairment notifications (68.8%) concerned male doctors. About three‐quarters of notifications about cognitive impairment (79.6%), physical illness (76.1%) and substance use (73.6%) concerned male doctors, as did 53.1% of notifications regarding mental illness (Table 4).

**TABLE 4:** Notifications of health impairment of medical practitioners to Australian health profession regulators, Australia (except New South Wales), 1 July 2012–30 June 2022, by notification category.^a^

Characteristic	Notification category			
	Mental illness	Physical illness	Cognitive impairment	Substance use
Number of reports	576	113	279	659
Sex				
Male	306 (53.1%)	86 (76.1%)	222 (79.6%)	485 (73.6%)
Female	270 (46.9%)	27 (23.9%)	57 (20.4%)	174 (26.4%)
Age group (years)				
Under 30	42 (7.3%)	6 (5.3%)	7 (2.5%)	19 (2.9%)
30–39	147 (25.5%)	12 (10.6%)	15 (5.4%)	182 (27.6%)
40–49	146 (25.3%)	18 (15.9%)	30 (10.8%)	182 (27.6%)
50–59	145 (25.2%)	23 (20.4%)	48 (17.2%)	179 (27.2%)
60–69	64 (11.1%)	29 (25.7%)	72 (25.8%)	69 (10.5%)
70 or older	32 (5.6%)	25 (22.1%)	107 (38.4%)	28 (4.2%)
Specialty				
General practice	180 (31.7%)	30 (27.0%)	126 (45.7%)	190 (29.0%)
Internal medicine	42 (7.4%)	8 (7.2%)	26 (9.4%)	35 (5.3%)
Surgery	21 (3.7%)	29 (26.1%)	34 (12.3%)	68 (10.4%)
Anaesthesia	23 (4.0%)	6 (5.4%)	14 (5.1%)	55 (8.4%)
Psychiatry	37 (6.5%)	7 (6.3%)	13 (4.7%)	21 (3.2%)
Other specialty	11 (1.9%)	6 (5.4%)	11 (4.0%)	28 (4.3%)
Non‐specialist	254 (44.7%)	25 (22.5%)	52 (18.8%)	259 (39.5%)
Missing data	8	2	3	3
Location (Modified Monash Model)				
Metropolitan	427 (75.2%)	85 (77.3%)	202 (73.5%)	473 (73.0%)
Regional	88 (15.5%)	16 (14.5%)	40 (14.5%)	84 (13.0%)
Rural or remote	53 (9.3%)	9 (8.2%)	33 (12.0%)	91 (14.0%)
Missing data	8	3	4	11
Country of training^a^				
Australia	422 (75.6%)	77 (75.5%)	165 (69.0%)	488 (78.2%)
Comparable jurisdictions	56 (10.0%)	12 (11.8%)	36 (15.1%)	72 (11.5%)
Non‐comparable jurisdictions	80 (14.3%)	13 (12.7%)	38 (15.9%)	64 (10.3%)
Missing data	18	11	40	35

^a^ Notifications regarding other types of health impairment (105 reports) are not included in this table.

Doctors aged 40–59 years comprised 30.5% of the medical workforce included in our analysis; 361 of 659 substance use notifications (54.8%) concerned doctors in this age group. Doctors aged 70 years or older comprised 3.4% of the medical workforce; 107 of 279 cognitive impairment notifications (38.4%) and 25 of 113 physical illness notifications (22.1%) concerned doctors in this age group (Tables 3 and 4).

Surgeons and anaesthetists comprised 9.1% of the medical workforce; 123 of 656 substance use notifications (18.8%) concerned doctors in these specialties. Rural and remote doctors comprised 8.3% of the medical workforce; 91 of 648 substance use notifications (14.0%) concerned doctors in these locations (Tables 3 and 4).

### 2.2 Sources and Outcomes

Health practitioners were the most frequent sources of health impairment notifications, including treating practitioners (166 notifications, 9.6%), other health practitioners (456, 26.3%) and self‐reports by practitioners (247, 14.3%) (Table 5). Of 256 notifications by employers, 101 were substance use notifications (39.5%); other agencies (e.g., police and prescription monitoring services) submitted 142 of 659 substance use notifications (21.6%). Physical health concerns were notified in 29 of 247 self‐reports (11.7%) and cognitive impairment in 34 self‐reports (13.8%).

**TABLE 5:** Characteristics of notifications of health impairment of medical practitioners to Australian health profession regulators, Australia (except New South Wales), 1 July 2012–30 June 2022.

Characteristic	Number
Health impairment notifications	1732
Source	
Treating practitioner	166 (9.6%)
Other health practitioner	456 (26.3%)
Self	247 (14.3%)
Employer	256 (14.8%)
Patient or their representative	273 (15.8%)
Other	334 (19.3%)
Type	
Voluntary	1196 (69.1%)
Mandatory	536 (30.9%)
Concern	
Substance use	659 (38.0%)
Mental health	576 (33.3%)
Physical health	113 (6.5%)
Cognitive impairment	279 (16.1%)
Other (not specified)	105 (6.1%)
Outcome^a^	
No further action	1035 (60.6%)
Referral to another body	15 (0.9%)
Non‐restrictive action e.g., caution, educational letter	81 (4.7%)
Voluntary undertaking	210 (12.3%)
Conditions	354 (20.7%)
Removal from practice	13 (0.8%)

^a^ Outcome was not available for 24 cases for which a final determination had not been reached by the time of data extraction.

A total of 536 health impairment notifications (30.9%) were mandatory notifications, including 380 (70.9%) from employers or other health practitioners (i.e., non‐treating colleagues). Mandatory notifications by treating practitioners accounted for 105 of 1732 health impairment notifications (6.1%), and there were fewer than 20 such notifications during each year of the study period.

By 30 June 2024 (2 years after the end of the study period), final determinations had been reached for 1708 of 1732 health impairment notifications (98.6%): 210 (12.3%) resulted in voluntary undertakings, and 367 (21.5%) in restrictions on or removal from practice (Table 5). The proportion of notifications resulting in restrictive action was higher for substance use notifications (186 of 643, 28.9%) than for other health concern categories (mental health: 112 of 568, 19.7%; cognitive impairment: 42 of 279, 15.1%; physical illness: 10 of 113, 8.8%; other: 17 of 105, 16.2%) (Table S1).

### 2.3 Factors Associated With Notifications

In multivariable analyses, the incidence of health impairment notifications was higher for male than for female doctors (aIRR, 1.45; 95% CI, 1.26–1.67) (Table 6).

**TABLE 6:** Characteristics of doctors and the incidence rate ratios of health impairment notifications (any or substance use): Multivariate logistic regression analysis.^a^

Characteristic	Incidence rate ratio (95% CI)	
	Any health impairment notifications	Substance use notifications
Sex		
Male	1.45 (1.26–1.67)	2.06 (1.60–2.64)
Female	1	1
Age group (years)		
Under 30	0.46 (0.34–0.61)	0.25 (0.15–0.41)
30–39	1	1
40–49	1.84 (1.51–2.24)	1.65 (1.22–2.24)
50–59	2.39 (1.94–2.94)	2.07 (1.51–2.85)
60–69	1.96 (1.57–2.44)	1.09 (0.76–1.58)
70 or older	2.92 (2.30–3.70)	0.92 (0.57–1.49)
Specialty		
Internal medicine	1	1
General practice	1.94 (1.52–2.49)	2.47 (1.56–3.89)
Surgery	1.76 (1.30–2.39)	2.43 (1.39–4.24)
Psychiatry	2.28 (1.62–3.21)	2.47 (1.31–4.66)
Anaesthesia	1.57 (1.12–2.19)	2.83 (1.66–4.83)
Other specialty	0.75 (0.52–1.08)	1.07 (0.57–2.01)
Non‐specialist	2.48 (1.91–3.22)	3.25 (2.00–5.27)
Location (Modified Monash Model)		
Metropolitan	1	1
Regional	1.33 (1.12–1.58)	1.14 (0.84–1.54)
Rural	1.27 (1.03–1.57)	1.47 (1.06–2.05)
Remote	1.55 (1.03–2.33)	2.16 (1.28–3.65)
Country of training		
Australia	1	1
Comparable jurisdictions	0.96 (0.79–1.16)	0.86 (0.62–1.21)
Non‐comparable jurisdictions	0.53 (0.43–0.64)	0.39 (0.29–0.54)

Abbreviation: CI, confidence interval.

^a^ Complete case data for all covariates (99,969 doctors and 724,525 observations during 2012–2022), adjusted for annual time‐varying year of registration.

The incidence of health impairment notifications increased with age group (70 years or older vs. 30–39 years: aIRR, 2.92; 95% CI, 2.30–3.70). The incidence of substance use notifications was highest for doctors aged 50–59 years (vs. 30–39 years: aIRR, 2.07; 95% CI, 1.51–2.85) (Table 6).

The incidence of health impairment notifications was higher for psychiatrists (aIRR, 2.28; 95% CI, 1.62–3.21), general practitioners (aIRR. 1.94; 95% CI, 1.52–2.49) and non‐specialists (aIRR, 2.48; 95% CI, 1.91–3.22) than for internal medicine physicians. The incidence of substance use notifications was highest for non‐specialists (vs. internal medicine physicians: aIRR 3.25; 95% CI, 2.00–5.27); among doctors with specialist qualifications, it was highest for anaesthetists (vs. internal medicine physicians: aIRR, 2.83; 95% CI, 1.66–4.83) (Table 6).

The incidence of health impairment notifications was higher for doctors in regional (aIRR, 1.33; 95% CI, 1.12–1.58), rural (IRR, 1.27; 95% CI, 1.03–1.57) and remote areas (aIRR, 1.55; 95% CI, 1.03–2.33) than in metropolitan areas. The incidence of substance use notifications increased with geographic remoteness (remote vs. metropolitan: aIRR, 2.16; 95% CI, 1.28–3.65) (Table 6).

The incidence of health impairment notifications was similar for doctors who trained in Australia or in comparable jurisdictions. The incidence was lower for doctors who qualified in non‐comparable jurisdictions than for those who trained in Australia, both overall (aIRR, 0.53; 95% CI, 0.43–0.64) and for substance use notifications (aIRR, 0.39; 95% CI, 0.29–0.54) (Table 6).

The sensitivity analysis restricted to health impairment notifications that resulted in regulatory action yielded broadly similar results. However, the differences between notification incidence for surgeons, psychiatrists and anaesthetists and that for internal medicine specialists were not statistically significant in the sensitivity analysis, nor were the differences by geographic location, possibly because of the smaller numbers of notifications included (Table S2).

## 3 Discussion

During 2012–2022, Ahpra received health impairment notifications for 1.1% of doctors. However, the overall prevalence of impairment among doctors in Australia remains unknown. Australian studies have found that 6% of doctors report current diagnoses of depression [23] and 15% potentially hazardous alcohol use [24]. Similarly, a large survey of doctors found that 15% met the diagnostic criteria for alcohol misuse or dependence [25].

As noted previously, illness does not necessarily indicate impairment, and only some unwell doctors are impaired in their ability to practise safely. Nevertheless, it seems unlikely that medical regulators are identifying all impaired doctors.

The lower incidence of health impairment notifications for female doctors may reflect lower rates of substance use among women [26, 27] and more frequent help‐seeking behaviour [28], increasing the likelihood that a condition is treated before it causes impairment. The incidence of health impairment notification was higher for doctors in rural and remote areas than in metropolitan areas. With increasing remoteness, the number of doctors per resident population declines, whereas on‐call demands and job complexity increase [29]. Our findings support ongoing efforts to alleviate the maldistribution of the medical workforce in Australia.

Ahpra more frequently received health impairment notifications from other health practitioners than from doctors reporting their own impairment. Relatively few health impairment notifications were submitted by patients or their representatives, in stark contrast to notifications about doctors' conduct and performance. It is possible that recognising impairment is aided by the personal and clinical knowledge of other health practitioners [30].

Mandatory notifications by treating practitioners were infrequent (6.1% of all mandatory notifications), and the number did not decrease substantially after the reporting threshold was raised in 2019 (fewer than 20 each year).

The incidence of specific health impairment notification types differed by specialty, possibly reflecting the nature of doctors' work, professional cultures and access to medications with potential for misuse. For example, the proportion of physical health notifications concerning surgeons, whose work is highly procedural and requires dexterity, was nearly five times the proportion of surgeons in the workforce. Further, the proportion of substance use notifications concerning anaesthetists, who have greater access to potentially addictive medicines [31], was twice as large as their proportion of the workforce. This finding is consistent with previous reports that anaesthetists are over‐represented among doctors with substance use disorders [32]. The proportion of mental health‐related notifications about psychiatrists, who may be drawn to the specialty by personal experiences [33] and who work in psychologically demanding roles with patients and colleagues attuned to signs of mental illness [34], was also twice that of their workforce proportion.

In multivariate analyses, sex, age, medical specialty and country of training also influenced the incidence of health impairment notifications.

We found that the incidence of health impairment notifications concerning non‐specialist doctors was higher than for other doctors. Non‐specialists may work in demanding roles with less job flexibility, autonomy and security, and this may lead to poorer health. Fear that health conditions could impede specialty training may delay non‐specialists seeking help. Alternatively, unsuccessful attempts to enter or complete specialty training could precipitate deterioration in doctors' health.

As life expectancy increases, the proportion of doctors aged 70 years or older is rising. Older doctors possess a depth of wisdom and experience that can benefit patients and the profession, but ageing can be associated with a decline in cognition, vision, dexterity and up‐to‐date clinical knowledge [35, 36, 37]. Our findings support efforts to facilitate career and retirement planning and the early identification of health concerns [38] without discouraging safe practice into higher age [7, 39].

Doctors who trained in health systems not comparable with that of Australia face numerous challenges [40]. They must navigate cultural differences and further examinations or other training hurdles, and often work in understaffed areas. However, we found that they were less frequently the subjects of health impairment notifications than doctors who trained in Australia or comparable jurisdictions. Possible explanations that should be investigated include a healthy migrant effect (whereby healthier and more motivated people are more likely to migrate to a new country) [41], cultural differences in the frequency of substance use, biases that lead to concerns about these doctors being attributed to poor conduct or competence rather than recognising health concerns or systematic barriers to recognising health impairment, including cultural stigmatisation of mental illness.

### 3.1 Limitations

Health impairment notifications are an imperfect marker of impairment. Some impaired doctors may not be reported to the regulator [15, 42], and reported cases may systematically differ from non‐reported cases because of reporting bias (e.g., related to ageism, racism or sexism) or ascertainment bias (e.g., impairment in sole practitioners may be less likely to be identified by colleagues). Conversely, some doctors who were subjects of health impairment notifications may have been practising safely, but the notification was vexatious or arose from a misunderstanding of reporting thresholds.

Our findings may not be generalisable to doctors practising in New South Wales, from where Ahpra does not receive or investigate notifications.

Health concerns were coded using Ahpra categories. We had no further health information about doctors. Where multiple health concerns were recorded, we selected one for the purposes of categorisation, with priority for organic causes of impairment. Consequently, health concerns may have been misclassified in some cases.

Further, as we had no information about the amount of time doctors worked, the number or the complexity of the needs of patients they saw, or practice type (solo or group practice), the influence of these factors could not be assessed.

## 4 Conclusions

Doctors with health conditions that are not adequately managed can be impaired in their medical practice, placing the public at risk of harm. At the same time, barriers to health care for doctors include fear of regulatory consequences [9]. Medical regulators around the world face the challenge of protecting the public while treating doctors with health impairments compassionately [12]. Solving this dilemma has been impeded by the paucity of information needed for effective interventions.

Our 10‐year study of data for more than 110,000 doctors helps provide such information. It is the largest in the world to examine the frequency, influencing factors and outcomes of health impairment notifications to a medical regulator.

Although health impairment notifications are infrequent, they are more frequent for psychiatrists, general practitioners and non‐specialists than for internal medicine physicians, for doctors in rural and remote locations than those in metropolitan areas and for male doctors; their incidence increases with age. The proportion of substance use notifications concerning anaesthetists was larger than their proportion of the medical practitioner workforce. These findings indicate that targeted strategies are needed to ensure that these groups of doctors have access to timely and trustworthy health care. How to best identify and intervene with older doctors whose fitness to practice is falling should be investigated further. The psychosocial hazards faced by doctors in specialties in which impairment is particularly frequent, including psychiatry, primary care and anaesthesia, and by doctors in rural and remote locations, also require attention.

## Author Contributions

**Marie M. Bismark:** conceptualisation, funding acquisition, methodology, supervision, writing (original draft). **Dilanka Hettiarachchi:** methodology, writing (review and editing). **Martin Fletcher:** conceptualisation, writing (review and editing). **Owen Bradfield:** writing (original draft). **Anu Tayal:** project administration, formal analysis. **Yamna Taouk:** supervision, methodology, formal analysis, writing (original draft).

## Funding

This study was funded by a National Health and Medical Research Council Investigator grant to Marie Bismark (34003).

## Conflicts of Interest

Martin Fletcher was chief executive officer of the Australian Health Practitioner Regulation Agency during the study period. No further relevant disclosures.

## Supporting information


**Data S1:** mja270131‐sup‐0001‐supinfo.pdf.

## Data Availability

This study did not generate original data.
