# Supplementary material for: Health Impairment Notifications About Doctors to the Australian Medical Regulator, 2012–2022: A Retrospective Cohort Study
Source: Med J Aust. 2026 Jan 14;224(1):e70131. doi: 10.5694/mja2.70131 (PMC12803957; doi:10.5694/mja2.70131)
Supplement: Supplementary file 1 — Data S1: mja270131‐sup‐0001‐supinfo.pdf. [file MJA2-224-0-s001.pdf]

The Medical Journal of Australia

Supporting Information

Supplementary results

This appendix was part of the submitted manuscript and has been peer reviewed. It is posted as supplied by the authors.

Appendix to: Bismark MR, Hettiarachchi D, Fletcher M, Bradfield O, Tayal A, Taouk Y. Health impairment notifications about doctors to the Australian medical regulator, 2012–22: a retrospective cohort study. *Med J Aust* 2026; doi: 10.5694/mja2.00000.

Supplementary results

Table S1. Notifications of health impairment of medical practitioners to Australian health profession regulators, Australia (except New South Wales), 1 July 2012 – 30 June 2022, for which final determinations had been reached by 30 June 2024: outcomes, by report type

Outcome of notification	Mental health	Physical illness	Cognitive impairment	Substance use	Other health impairment
All notifications	576	113	279	659	105
All notifications with final determinations*	568	113	279	643	105
No further action	356 (62.7%)	88 (77.9%)	195 (69.9%)	329 (51.2%)	67 (63.8%)
Referral to another body	19 (3.3%)	3 (2.7%)	8 (2.9%)	50 (7.8%)	16 (15.2%)
Voluntary undertaking	81 (14.3%)	12 (10.6%)	34 (12.2%)	78 (12.1%)	5 (4.8%)
Conditions/removal from practice	112 (19.7%)	10 (8.8%)	42 (15.1%)	186 (28.9%)	17 (16.2%)

*Outcome not available for 24 cases for which a final determination had not been reached by the time of data extraction.

Table S2. Characteristics of doctors and the incidence rate ratios of health impairment notifications (any or substance use): sensitivity multivariate logistic regression analysis restricted to notifications that resulted in regulatory action

Characteristic	Adjusted incidence rate ratio (95% confidence interval)
Sex	
Male	1.27 (1.02–1.60)
Female	1
Age group (years)	
Under 30	0.39 (0.25–0.60)
30–39	1
40–49	1.41 (1.02–1.95)
50–59	2.32 (1.65–3.26)
60–69	1.49 (1.02–2.17)
70 or older	1.80 (1.15–2.82)
Speciality	
Internal medicine	1
General practice	1.61 (1.07–2.44)
Surgery	1.16 (0.66–2.02)
Psychiatry	1.46 (0.78–2.74)
Anaesthesia	1.67 (0.96–2.91)
Other specialty	0.45 (0.16–1.27)
Non-specialist	2.49 (1.62–3.84)
Location (Modified Monash Model)	
Metropolitan	1
Regional	1.28 (0.95–1.71)
Rural	1.20 (0.84–1.71)
Remote	1.43 (0.69–2.98)
Country of training	
Australia	1
Comparable jurisdictions	0.79 (0.55–1.13)
Non-comparable jurisdictions	0.53 (0.38–0.73)

STROBE Statement: Checklist of items that should be included in reports of cohort studies

Note: The page numbers in this checklist refer to the submitted manuscript, not to the published article or its Supporting Information file

	Item No	Recommendation	Page No
Title and abstract	1	(a) Indicate the study's design with a commonly used term in the title or the abstract	1
		(b) Provide in the abstract an informative and balanced summary of what was done and what was found	2
Introduction			
Background/rationale	2	Explain the scientific background and rationale for the investigation being reported	4
Objectives	3	State specific objectives, including any prespecified hypotheses	4
Methods			
Study design	4	Present key elements of study design early in the paper	5
Setting	5	Describe the setting, locations, and relevant dates, including periods of recruitment, exposure, follow-up, and data collection	5
Participants	6	(a) Give the eligibility criteria, and the sources and methods of selection of participants. Describe methods of follow-up	5
		(b) For matched studies, give matching criteria and number of exposed and unexposed	N/A
Variables	7	Clearly define all outcomes, exposures, predictors, potential confounders, and effect modifiers. Give diagnostic criteria, if applicable	6
Data sources/ measurement	8	For each variable of interest, give sources of data and details of methods of assessment (measurement). Describe comparability of assessment methods if there is more than one group	6,7
Bias	9	Describe any efforts to address potential sources of bias	5,6
Study size	10	Explain how the study size was arrived at	8
Quantitative variables	11	Explain how quantitative variables were handled in the analyses. If applicable, describe which groupings were chosen and why	6,7
Statistical methods	12	(a) Describe all statistical methods, including those used to control for confounding	7
		(b) Describe any methods used to examine subgroups and interactions	7
		(c) Explain how missing data were addressed	7
		(d) If applicable, explain how loss to follow-up was addressed	N/A
		(e) Describe any sensitivity analyses	7
Results			
Participants	13	(a) Report numbers of individuals at each stage of study—eg numbers potentially eligible, examined for eligibility, confirmed eligible, included in the study, completing follow-up, and analysed	8
		(b) Give reasons for non-participation at each stage	8
		(c) Consider use of a flow diagram	Figure 1
Descriptive data	14	(a) Give characteristics of study participants (eg demographic, clinical, social) and information on exposures and potential confounders	Table 1
		(b) Indicate number of participants with missing data for each variable of interest	Table 1, Table 2, Table 3

		(c) Summarise follow-up time (eg, average and total amount)	8
Outcome data	15	Report numbers of outcome events or summary measures over time	8
Main results	16	(a) Give unadjusted estimates and, if applicable, confounder-adjusted estimates and their precision (eg, 95% confidence interval). Make clear which confounders were adjusted for and why they were included	Table 4
		(b) Report category boundaries when continuous variables were categorized	Table 4
		(c) If relevant, consider translating estimates of relative risk into absolute risk for a meaningful time period	N/A
Other analyses	17	Report other analyses done—eg analyses of subgroups and interactions, and sensitivity analyses	10
Discussion			
Key results	18	Summarise key results with reference to study objectives	10
Limitations	19	Discuss limitations of the study, taking into account sources of potential bias or imprecision. Discuss both direction and magnitude of any potential bias	12
Interpretation	20	Give a cautious overall interpretation of results considering objectives, limitations, multiplicity of analyses, results from similar studies, and other relevant evidence	10,11,12
Generalisability	21	Discuss the generalisability (external validity) of the study results	12
Other information			
Funding	22	Give the source of funding and the role of the funders for the present study and, if applicable, for the original study on which the present article is based	N/A
